# Splicing heterogeneity: separating signal from noise

**DOI:** 10.1186/s13059-018-1467-4

**Published:** 2018-07-09

**Authors:** Yihan Wan, Daniel R. Larson

**Affiliations:** Laboratory of Receptor Biology and Gene Expression, Center for Cancer Research, National Cancer Institute, Bethesda, MD 20892 USA

## Abstract

Single-cell analyses have revealed a tremendous variety among cells in the abundance and chemical composition of RNA. Much of this heterogeneity is due to alternative splicing by the spliceosome. Little is known about how many of the resulting isoforms are biologically functional or just provide noise with little to no impact. The dynamic nature of the spliceosome provides numerous opportunities for regulation but is also the source of stochastic fluctuations. We discuss possible origins of splicing stochasticity, the experimental approaches for studying heterogeneity in isoforms, and the potential biological significance of noisy splicing in development and disease.

## Introduction

In recent years, there has been substantial progress in the development of methodologies to interrogate gene expression in single cells. Single-cell imaging has historically been the workhorse technology for such studies, but applications such as single-cell sequencing have rapidly advanced, with recent publications drawing conclusions from tens of thousands of individual cells [1–4]. The picture that emerges from these studies is that gene expression varies from cell to cell. These differences can be both genetic and non-genetic, and they can be stable or dynamic. Differences can arise from programmed specialization during development or through random processes that occur in the cell. Even at the mRNA level, abundance, sequence, and chemical modifications can vary among transcripts that are produced from the same sequence of DNA. Making sense of this variation has become an immense experimental and theoretical challenge.

The process of RNA synthesis leads to variation in mRNA abundance, which has been studied extensively [5]. However, RNA processing, specifically pre-mRNA splicing, has the potential to be an equally important source of variability in gene expression. Since the first discovery of splicing 40 years ago [6–8], accumulating knowledge about the spliceosome’s assembly and enzymatic mechanism, about the process of splice site selection, and on the coupling with transcription depicts a complex, multi-step, dynamic model involving a massive molecular machine. Each of these steps in splicing is subject to regulation, leading to the amazing diversity of alternatively spliced transcripts in virtually every organism in which RNA splicing is present. Each of these steps is, however, also subject to random fluctuations. Like all reactions that occur at the molecular level and rely on small numbers of molecules, stochastic (i.e., random) effects are the rule rather than the exception. This phenomenon was evident in the earliest observations of alternative splicing using chromatin spreads of the *Drosophila* chorion gene. In the same transcription unit, two alternative splicing isoforms were observed at the single-molecule level [9]. Since then, the proportion of transcripts that show alternative splicing has asymptotically approached 100%. From ‘a list of genes’ in the 1980s [10], to ‘74% of all genes’ inferred from expressed sequence tag (EST)–genomic alignments and microarrays [11, 12], to ‘98–100% of multi-exonic genes’ in the next-generation sequencing era [13–16]. Single-cell sequencing has now revealed that splicing variability exists among tissues and between individuals [17–20].

Which transcripts are functional? How do we detect meaningful changes not only in alternative splicing but also in RNA editing or alternative poly-adenylation? And what experimental and conceptual advances will be needed for the next stage of research? In this special issue, new techniques and datasets are presented that are at the forefront of RNA biology. Here, we focus on the current understanding of variability in RNA processing, mostly on splicing. We hope to frame the following questions. 1) Where does splicing stochasticity come from? 2) How do we measure splicing variability? 3) What is the biological significance of splicing heterogeneity?

## Noise in splicing: where does it come from?

**Fig. 1:**
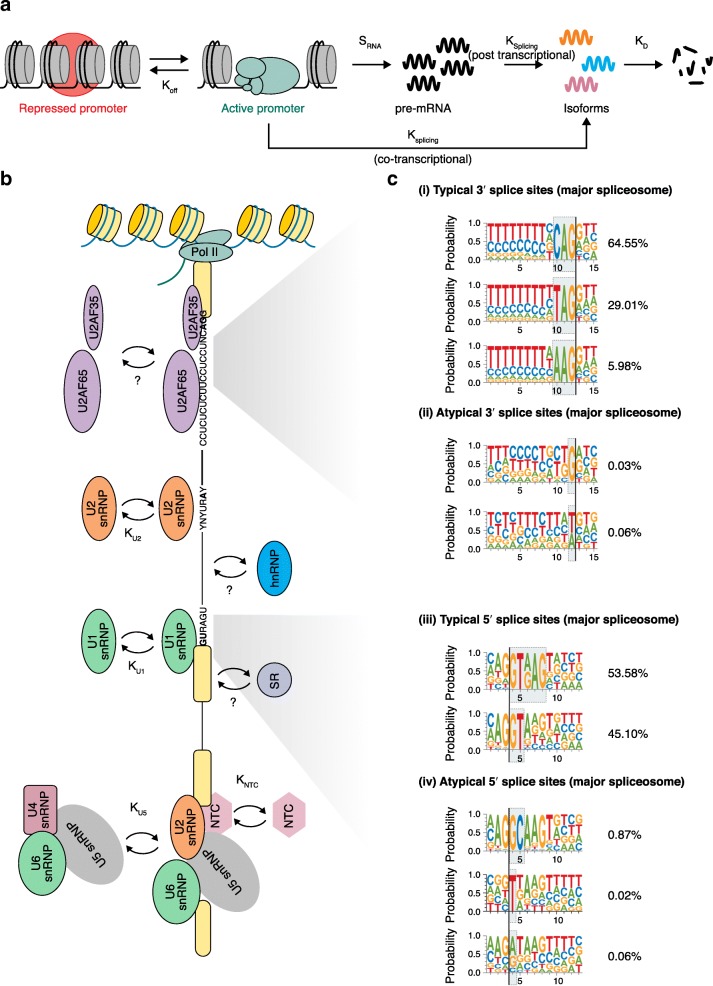
Stochastic events in splicing. The spliceosome is a single-turnover enzyme that assembles and disassembles for each splicing event. Splicing consists of a complex sequence of steps, and each step represents several biochemical reactions. These reactions involve binding and dissociation events, which include random variables at the molecular level. **a** Schematic representation of the steps associated with mRNA production: transition of the promoter between a repressed and an active state, transcription, co-transcriptional or post-transcriptional splicing to create heterogenous isoforms and mRNA degradation. **b** Kinetic scheme for co-transcriptional spliceosome assembly. The formation of the catalytically competent spliceosome starts with splice site recognition, which is a highly dynamic process. Although the in vivo measurements of snRNP kinetics are still missing, in vitro experiments provide evidence for the reversible binding of almost all of the major subcomplexes to the nascent RNA (e.g., the pairing between U1 and 5′ss, U2 and branchpoint, the binding of tri-snRNP and NTC are in a kinetic range of k = 0.13–0.35 min^− 1^). The binding dynamics between the U2AF complex and poly-pyrimidine tract and 3′ss are still poorly understood. The binding of heterogeneous nuclear ribonucleoproteins (hnRNPs) and SR proteins also regulates splicing dynamics. Their kinetics need to be further explored. **c** Variability of splice sites in the human genome. (i) Consensus motifs of the U2-type 3′ splice sites with AG at the border. (ii) Non-canonical motifs of U2-type 3′ splice sites with dinucleotides other than AG at the border. (iii) Consensus motifs of the U1-type 5′ splice sites with GT at the border. (iv) Non-canonical motifs of U1-type 5′ splice sites with dinucleotides other than GT at the border. *NTC* NineTeen complex, *Pol II* RNA polymerase II, *snRNP* small nuclear ribonucleoprotein

To understand the source of stochastic variability, a comparison with transcription is illuminating. One major source of variability in mRNA abundance is the time-varying activity of RNA synthesis called transcriptional ‘bursting’. Periods of active RNA synthesis are punctuated by long inactive periods [21–23]. The properties of the bursts are determined by *cis*-acting elements such as enhancers [24–27] and promoters [28–30], and by *trans*-acting activators [31, 32] and chromatin remodelers [33–35]. In particular, the initiation of RNA synthesis is the major source of variability, with downstream processes such as elongation, cleavage, release, and termination contributing little. Notably, enhanceosomes and pre-initiation complexes assemble and dissemble within a timescale of seconds [36, 37], and a ‘successful’ event results in the production of a transcript with low efficiency (from about 10% of complexes formed) [38–40]. Similarly, splicing is also a dynamic process that relies on both *cis*-acting elements and *trans*-acting modulators [41]. The assembly and disassembly of the spliceosome E complex occurs at a timescale of seconds to minutes [42]. The spliceosome is also a single turnover enzyme that disassembles after the completion of each splicing event (Fig. 1). Thus, the spliceosome would need to assemble and disassemble dozens of times (or more) during the production of any one transcript. The assembly of a spliceosome is determined by information residing in the consensus branch point and 5′, 3′ splice sites, but it can be affected by multiple levels of regulation, such as activities of silencer or enhancer sequences, the binding of SR proteins or heterogeneous nuclear ribonucleoproteins (hnRNPs), transcriptional kinetics, nucleosome positioning, and DNA template or chromatin modifications [15, 43]. When attempting to understand splicing noise, we can begin by looking at the composition and kinetics of the splicing machinery (Fig. 1).

For each nascent RNA molecule generated from transcription, the spliceosome needs to first recognize the correct splice sites, then assemble to complete intron removal and exon ligation, and then disassemble. Intron and exon definition is the key step in the initiation of spliceosome assembly. The 5′ splice site consensus AG|**GU**RAGU is present at the exon–intron junction. The 15-nucleotide 3′ splice site Y_10_NC**AG**|G is present at the intron–exon junction. At a variable distance upstream of the 3′ splice site (10–50 nucleotides (nt) for human transcripts) is the branch point consensus YNYUR**A**Y [44–47]. The dinucleotide pair GU-AG is present in over 98% of all intron sequences that are removed by the spliceosome, but variations are found in neighboring bases [48, 49] (Fig. 1c).

Randomness is generated by several aspects of this splice-site-recognition step. First, the sequence information from the nascent RNA transcripts is ambiguous and highly degenerate, especially in mammals. The intron or exon definition step requires spliceosomes to read the information from more than ~ 30 bases accurately [50]. This recognition mostly relies on the base-pairing between U1 and U2 small nuclear RNAs (snRNAs) and the nascent RNA, but RNA modifications and bulged nucleotides make this base-pairing highly flexible [49, 51]. Sequence alone is not sufficient allow the accurate identification of splicing boundaries, even for short introns (≤ 134 bp) in human transcripts [52]. Moreover, many sequences in the mammalian genome match the consensus but are not recognized as real splice sites and the mechanism behind this discrimination is poorly understood. Second, mutations and single nucleotide variants (SNVs) in the template sequence generate moving targets for the spliceosome. Millions of genetic variants in the human genome have been uncovered through the 1000 Genomes project [53]. Multiple methods, such as machine learning [54], splicing quantitative trait loci (QTL) [55], and integrative genome-wide association studies (iGWAS) [56] have revealed that SNVs are associated with alternative splicing. These SNVs could change the splice sites directly or could alter a splicing regulatory sequence. Furthermore, the long introns in human transcripts also provide ample mutational opportunities for the creation of new or weak splice sites and for the generation of new exons (exonization) [20, 57]. Third, this ‘reading and recognition’ process is coordinated by splicing enhancer and silencer sequences through recruitment of SR proteins and hnRNPs [58]. Binding motifs for SR proteins and hnRNPs can be found in the majority of exons and introns [59, 60]. The role of RNA-binding proteins (RBP) can be synergic or competitive. The output of a splicing event will be affected by the motif sequence of the pre-mRNA and the array of RBP concentrations in the cell.

The complexity in the template pre-mRNA brings a primary source of stochasticity, even before considering the assembly of the spliceosome itself. The spliceosome consists of hundreds of proteins and multiple snRNAs. Initially, splicing ‘commitment’ was thought to occur once the intron–exon boundary has been defined [61]. Recent studies have revealed, however, that the spliceosome is a highly flexible and reversible enzyme. Spliceosome assembly can be initiated by either a U1- or a U2-first pathway [62]. After assembly initiation, the spliceosome can switch between different catalytic conformations that favor forward or reverse progress [41]. The splicing catalytic process is iso-energetic and driven by numerous ATPases, resulting in two transesterification processes that are both reversible in the proper ionic environment [63, 64]. Recent single-molecule research on spliceosome assembly has revealed that almost all of the steps in splicing are reversible [65, 66]. In the context of a highly flexible and reversible spliceosome assembly process, the alternative splicing decision may be the result of kinetic competition between different spliceosome assembly pathways.

**Fig. 2:**
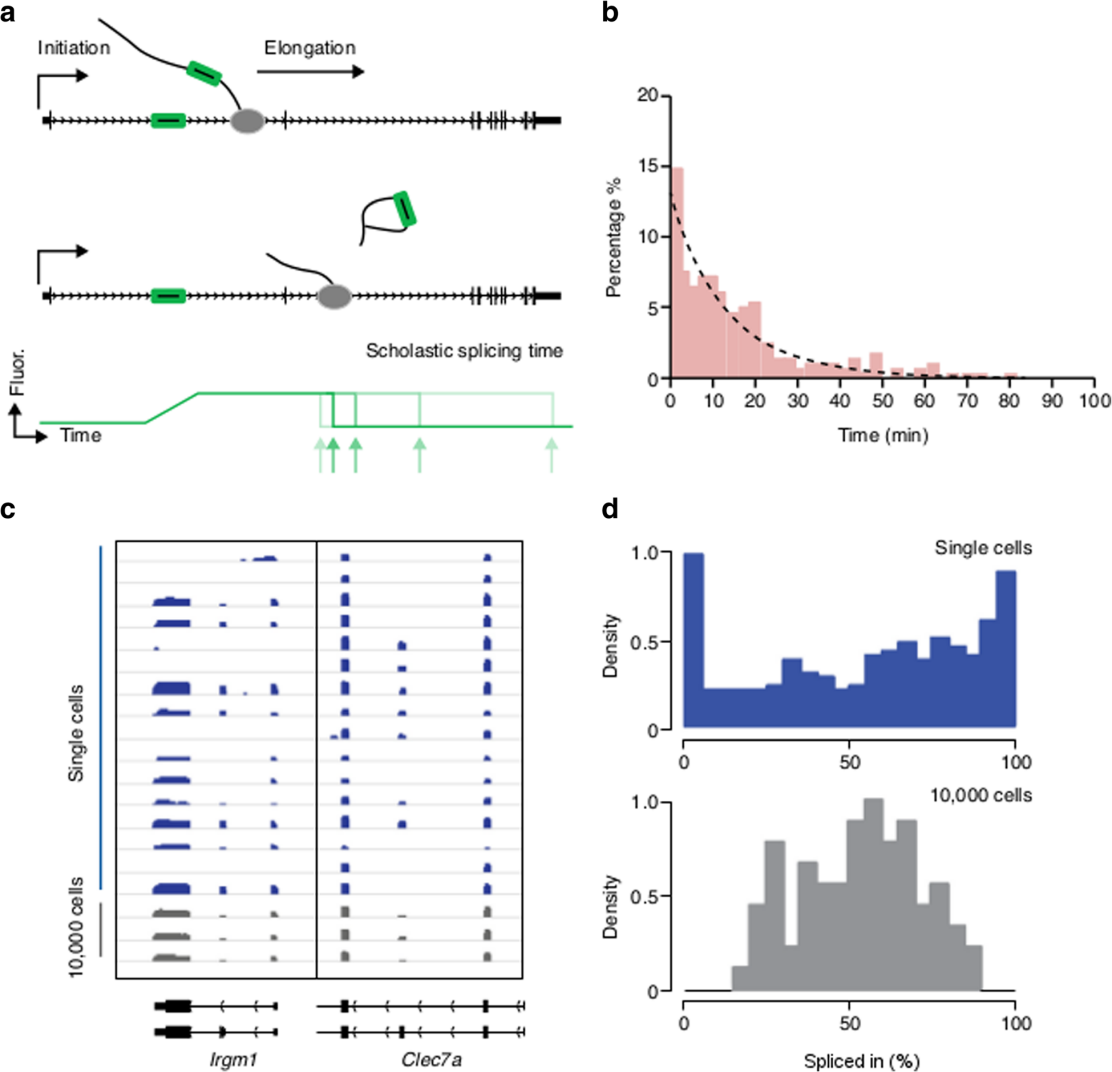
Single-cell or single-molecule measurement reveals the stochasticity in splicing kinetics and splice-site choice. **a** Schematic of stochasticity in splicing kinetics. By labeling the intron with MS2 stem loops (*green*), the splicing stochasticity can be recorded through the fluorescence fluctuations at the transcription site. **b** In this histogram, the splicing kinetics of a gene exhibit an exponential distribution, indicating a stochastic process. In this simple stochastic scenario, the most likely splicing time is the shortest measurable time. **c** Comparing RNA-seq reqd densities from single cells (*blue*) to a population of cells (*gray*). Two representative genes, *Irgm1* and *Clec7a*, each with two splicing isoforms (*bottom*) are shown. Single cell RNA-seq revealed distinct splicing patterns in individual cells. **d** Distribution of exon inclusion ratios (Percent Spliced in (PSI) scores, x-axis) for alternatively spliced exons in single cells (*blue*) and population cells (*gray*). Single-cell RNA-seq reveals a bimodal distribution of splicing isoforms, which is otherwise measured as a normal distribution with different splicing efficiencies from population cells

Years of work on transcription have solidified the view that heterogeneity is a dynamic phenomenon: a gene may appear to be ‘off’, only to be expressed again minutes or hours later. Likewise, understanding splicing stochasticity requires an understanding of splicing dynamics. Splicing can be viewed as a process that is affected by multiple kinetic variables: 1) transcription kinetics affecting the generation of nascent RNAs; 2) the diffusion kinetics and assembly dynamics of the macromolecules involved in recognizing splice sites; 3) the spliceosome catalytic dynamics. Determining these kinetic parameters in vivo and how they work in concert are important for understanding splicing stochasticity. To address this question, a simple starting point is the time-lapse measurement of the splicing output—the generated mRNA isoforms. Population-level measurements that are based on time-resolved nascent-RNA sequencing and quantitative real-time PCR (RT-PCR) elucidate average splicing times ranging from 5 to 14 min in mammalian cells and of less than 5 min in yeast [67–69]. Nevertheless, the average measurement of a cell population may not reflect the behaviors of individual cells. Live-cell fluorescence microscopy based on a set of reporter genes labeled by MS2 and/or PP7 stem loops (Fig. 2a) probes the splicing kinetics at the single-cell level and reveals variable timescales (for example, from 20 s to many minutes) [70–72]. Single-molecule intron tracking (SMIT) combined with long-read sequencing showed that splicing can take place before the RNA polymerase transcribes even a few dozen nucleotides downstream of the 3′ splice site (i.e., a few seconds given the polymerase transcribing rate) in yeast [73].

One explanation for the lack of consensus across the various methods used might be the difficulties in detecting the whole dynamic range of splicing. For example, if splicing time is exponentially distributed over a broad range (Fig. 2a, b), the measured time will depend on the time resolution of the method. Imaging or pulse-chase methods might overestimate the duration of very short events or might underestimate the duration of extremely long events. Likewise, for steady-state biochemical methods, the inferred dynamic parameters rely on the assumption that all intermediates are identified and analyzed, whether they are on chromatin or in the nucleoplasm.

Above all, the stochasticity of splicing could result in variability in both splice site selection and splicing kinetics. How do splicing kinetics associate with splice site selection? Does alternative splicing exhibit different kinetics? Evidence is emerging that, at least for certain genes, alternative splicing occurs mostly post-transcriptionally, whereas constitutive exons are spliced co-transcriptionally [74]. In addition, changing the nucleotides next to GU at the 5′ splice site can alter both the kinetics of spliceosome remodeling and splicing efficiency [75]. How the spliceosome makes a choice amongst splice sites during the kinetic competition between splicing and transcription [70, 71, 74, 76] is still an unanswered question that requires further investigation.

## Can we measure the extent of stochastic RNA processing experimentally?

The initial concept of ‘splicing noise’ comes from the analysis of EST sequences and microarray-based mRNA abundance measurements [77]. These data suggested a positive correlation between the number of alternative isoforms and the number of splicing reactions (i.e., the number of introns per gene and the level of gene expression). A more precise evaluation was provided by the de novo identification of splice junctions based on RNA-seq data. Such studies revealed the existence of a large class of low-abundance isoforms [78]. Most of these isoforms contain the GU-AG dinucleotides, which indicate that they are generated from a random splice site choice. When thousands of independent RNA-seq datasets were combined, a significant number of previously unannotated splice junctions became evident across different tissues and cell types [79]. Although the current focus when analyzing these data is still on major alternative splicing events, a more comprehensive analysis across all splicing junctions would be beneficial for elucidating the distribution of isoform frequencies. Interestingly, a simple two-parameter Weibull distribution can be used to explain the statistical distribution of the isoforms of all transcribed genes, indicating a possible general model of stochastic splicing [80].

Ideally, measurement of the stochasticity in splicing requires capturing each individual event in a population. Single-cell RNA-seq [3, 81–87] provides a promising avenue, but there are two major challenges: the first comes from the single-molecule capture efficiency. Using a spike-in assisted evaluation, Wold and colleagues [88] were able to provide an estimate of single-molecule capture efficiency of around 0.1, meaning that rare events are not represented in the single-cell sequencing library. The second challenge is to distinguish the biological stochasticity from technical noise, which is an enduring issue in single-cell analysis. Careful evaluation of the technical noise with quantitative statistical methods is necessary. Two recent studies carried out splicing analysis at the single-cell level [89, 90]. One unexpected discovery is that about 20% of the genes exhibit a bimodal distribution of certain splicing isoforms (Fig. 2c, d). These bimodal genes are related to differentiation and cell-type determination. After excluding technical artifacts caused by a low capture rate, there are two possible explanations for the bimodal distribution. First, the distribution may be due to extrinsic noise. For example, heterogeneity in the concentration of splicing regulators in different cells might result in the same pre-mRNA being processed differently. Second, the bi-modality might be caused by intrinsic noise. For example, in transcription, slow promoter kinetics will result in a bimodal distribution of gene expression [91]. Similarly, a slow transition parameter in isoform processing could also generate a bimodal distribution of isoforms in a cell population.

Single molecule long-read sequencing (Pacbio RNA-seq, iso-seq) [92, 93] is another promising technique for surveying isoform diversity. It can provide confident high-quality reads for transcripts over 20 kb, and over 10% of novel splice junctions have been identified through this strategy. The drawbacks are low throughput (i.e., limited reads per SMRT cell) and the potential for relatively high error rates in long reads.

Single-cell sequencing is comprehensive but suffers from low sensitivity and the potential for the introduction of error during library preparation and analysis. Single-molecule imaging is a complementary method. Single-molecule fluorescent in situ hybridization (smFISH) [94, 95] is a powerful way to quantify the absolute abundance of endogenous RNA transcripts in individual cells. Alternative splicing can be visualized by detecting the unique sequences of the different isoforms. The major advantage of this method compared to single-cell RNA-seq is that it provides both spatial information and sequence-specific information. For example, by probing the introns undergoing alternative splicing in the genes Sxl and nPTB, Vargas et al. [74]showed that alternatively processed introns have delayed kinetics and are more frequently detected in the nucleoplasm. Waks et al. [96] probed the alternative spliced exons in genes CAPRIN1 and MKNK2, and examined the cell-to-cell variability by measuring the fraction of isoform abundance. Notably, they found that the distribution of isoform ratio could be explained by a theoretical stochastic model [96]. Nevertheless, standard smFISH requires the targeting of a single transcript with probes of approximately 48 oligonucleotides, each spanning about 17–22 nt and labeled at their 3′ end with one fluorophore. For the large majority of alternatively spliced isoforms, which only have slight differences in their mRNA sequences, a more sensitive approach such as the recently developed inosine fluorescence in situ hybridization (inoFISH) [97] is necessary.

Both smFISH and inoFISH require killing cells, and neither addresses the dynamic nature of splicing. To explore the stochasticity in splicing, it is necessary to record splicing kinetics in living cells. Taking advantage of the bacteriophage MS2 stem-loop and fluorescence-labeled coat proteins, researchers now can record RNA dynamics at the single-molecule level. Initially, fluorescence recovery after photobleaching (FRAP) together with MS2 stem-loop-labeled genes were used to monitor splicing and transcription kinetics [98]. The improvement in the imaging and analysis of RNAs at the single-molecule level enabled the direct observation of nascent RNAs at the gene locus. The fluctuation of intron and exon signals was recorded, and transcription and splicing kinetics were extracted through the cross-correlation function [70, 72]. With the advance of genome editing [99, 100], it is now possible to label single molecules of RNA produced from endogenous loci, which will allow tracing of the nascent RNA synthesized under physiological conditions. The information provided by live imaging of splicing of endogenous genes will extend our understanding of the stochasticity in splicing kinetics, including the impact of signaling networks and the chromatin environment.

Tremendous progress in the single-cell sequencing and real-time measurement of single-molecule fluorescence has accelerated our understanding of splicing stochasticity. An integrated method that combines the ‘bird’s-eye view’ provided by high-throughput sequencing and the detailed information from time-lapse single-molecule microscopy will facilitate further advancements.

## Understanding the physiological role of noise in RNA processing

To understand a potential functional role for variability (stochastic or otherwise) in RNA sequence, a potential starting point is the assessment of the protein products. The proposition of ‘one gene, multiple proteins’ is rooted in the early days soon after the discovery of alternative splicing. Yet, there is debate on the extent to which alternative splicing can change the protein reservoir. Of course, there are numerous examples showing that functionally distinct proteins are generated from alternative splicing isoforms. More recently, using ribosome profiling, it has been shown that more than 75% of medium-to-high abundance alternative cassette exons are occupied by ribosomes [101]. Over 60% of these cassette exons preserve the reading frame, in agreement with the observation that short, frame-preserving cassette exons are more evolutionarily favored [102]. An opposing view is that although thousands of alternative splicing isoforms are identified through RNA-seq, only a small portion of them are identified by large-scale mass spectrometry [103]. In the early days of GENCODE, Tress et al. [104] examined the limited number of reported alternative splicing events. They concluded that many alternative spliced transcripts, if translated, would drastically change the structure and function of the protein products. Nevertheless, it is hard to predict the protein structure that would result from some isoforms, or whether the sequence would result in an unstable folding status [104]. The follow-up study, based on a large-scale human proteomics database analysis, suggests that most highly expressed genes have one dominant isoform [105]. Nevertheless, owing to the limited sensitivity of mass spectrometry-based proteomics, we still do not know what proportion of alternative splicing isoforms will result in functional proteins.

Did biological systems evolve to suppress splicing noise? Alternatively, has the system evolved to exploit this noise? The most common noise-reducing regulatory mechanism is negative feedback. RNA quality control systems, such as nonsense-mediated decay (NMD), nonstop decay (NSD), and no-go decay (NGD), have evolved to mitigate errors in RNA processing [106]. In addition to negative feedback, kinetic proofreading also plays a role in dampening splicing noise [107, 108]. On the other hand, noisy splicing has been proposed to give rise to population heterogeneity and may be essential in neurogenesis [109, 110], innate immunity [111], and evolution [112, 113]. Notably, recent work has also demonstrated a global alteration in splicing in cancers that involve mutations in core spliceosomal subunits such as U2AF1 and SF3B1 [114]. Intensive sequencing efforts from patients’ samples argued that the splicing changes in these patients are minor and highly variable [115–117]. To date, it has been difficult to attribute either the cancer phenotype or the prognosis to isoform changes affecting a specific set of genes. Cancer is an evolutionary disease and these spliceosomal mutations often occur at an early stage [118–120]. One possibility might be that the mutations in spliceosomal proteins function as an amplifier of splicing noise, as has been suggested for splicing alterations in other disease states [121]. Low-abundance isoforms that are generated through splicing noise may allow the new variant to be evolutionarily tested and could benefit tumor progression in a heterogeneous way.

## Current limitations and outlook

Splicing has been studied intensively, but it is only one of the processes that determine the chemical composition of mRNA. The roles of RNA editing and RNA modifications are now coming into focus as additional potential sources of heterogeneity. Transcriptome profiling techniques are powerful because of the exquisite detail they provide, and imaging allows researchers to follow cells over time. Future efforts to combine these advantages in order to generate longitudinal studies of transcription and splicing are promising but in the early stages [122]. In the meantime, the problem of interpreting the phenotypic consequences of variability remains a considerable challenge.

## Abbreviations

EST: Expressed sequence tag
hnRNP: Heterogeneous nuclear ribonucleoprotein
inoFISH: Inosine fluorescence in situ hybridization
RBP: RNA-binding protein
smFISH: Single molecule fluorescent in situ hybridization
snRNA: Small nuclear RNA
SNV: Single nucleotide variant
